# Regulatory natural autoantibodies suppress inflammation and SLE disease activity

**DOI:** 10.1186/ar3961

**Published:** 2012-09-27

**Authors:** GJ Silverman, J Vas, C Grönwall

**Affiliations:** 1 NYU School of Medicine, New York, NY, USA

## Background

Our recent studies have shown that IgM natural antibodies (NAbs) that recognize epitopes on apoptotic cells (ACs) are present from birth and can have potent immunoregulatory properties. These antibodies, by recruiting early complement recognition factors, C1q or mannose-binding lectin, enhance AC clearance by innate immune cells and also mediate suppression of proinflammatory responses induced by Toll-like receptor (TLR) agonists [1,2]. Furthermore, *in vivo *administration blocks the development of inflammatory autoimmune disease in experimental murine models [2]. We therefore explored the mechanistic basis for these properties and assessed for potential clinical relevance.

## Methods

Bone-marrow-derived myeloid DCs and macrophages were stimulated with TLR agonists or with immune complexes (ICs) composed of IgG-autoantibody-chromatin or IgG-autoantibody-RNA. We then evaluated production of inflammatory cytokines, and expression of co-stimulatory molecules, which are markers of DC activation. MAPK activation was evaluated by phospho-flow and immunofluorescence microscopy. In a cohort of 120 SLE patients, we examined the relationships between levels of IgM natural autoantibodies to apoptosis-associated antigens, with lupus-associated autoantibodies and features of disease.

## Results

In mechanistic studies, during responses to TLR agonists, anti-AC IgM decreased DC activation of the primary MAPKs; ERK1/2, JNK, and particularly p38. This inhibitory response was linked to anti-inflammatory signaling events dependent on nuclear localization of MAPK phosphatase-1, a factor known to also mediate glucocorticoid suppression of immune responses. Anti-AC NAb IgM also suppressed both DNA-IC-induced and RNA-IC-induced cytokine production, and upregulation of CD86 and CD40 on DCs. IgM anti-AC NAb also suppressed IC-mediated MAPK activation [3]. In clinical surveys, IgM autoantibodies to apoptotic cell membranes were commonly detected in healthy humans and SLE patients. In SLE patients, we found that IgM anti-AC levels were significantly higher in patients with low disease activity and less organ damage (by SELENA-SLEDAI, the physician's evaluation and the SLICC damage score). Furthermore, IgM anti-AC levels were also significantly higher in patients without cardiovascular events. In contrast, IgM anti-cardiolipin and IgM anti-dsDNA were significantly higher in patients without renal disease [4].

## Conclusion

Our recent studies have demonstrated a direct inhibitory effect of the NAb IgM on inflammatory responses induced by IgG-nucleic acid ICs. Our clinical surveys also contribute to emerging evidence that regulatory anti-AC NAbs may oppose the influence of pathogenic lupus autoantibody-ICs and thereby play roles in maintaining immune homeostasis. These results support the hypothesis that some IgM autoantibodies are part of a natural immune repertoire that provides protection from certain clinical lupus features.
